# Conservative management of primary malignant melanoma of the bladder: a case report

**DOI:** 10.1186/s13256-020-02602-7

**Published:** 2021-02-05

**Authors:** Sebastiano Rapisarda, Maida Bada, Andrea Polara, Felice Crocetto, Massimiliano Creta, Francesco Chiancone, Massimo Occhipinti, Rossella Bertoloni, Armando Marciano, Luca Aresu, Arjan Nazaraj, Sara Grosso, Gaetano Grosso

**Affiliations:** 1 Department of Urology, Hospital Pederzoli, Peschiera del Garda, Verona (VR) Italy; 2 Department of Urology, Hospital S.Bassiano, Bassano del Grappa, VI Italy; 3 Department of Neurosciences, Reproductive and Odontostomatological Sciences, Urology and Andrology Unit, University of Naples Federico II, Via Pansini No. 5, 80131 Naples, Italy; 4 Department of Urology, AORN A. Cardarelli, Naples, Italy

**Keywords:** Bladder cancer, TURB, Bladder melanoma, Cystectomy, BCG

## Abstract

**Background:**

Primary malignant melanoma (PMM) of the bladder represents a very rare clinic-pathologic entity. Given the rarity of the disease, the best treatment option is not well recognized.

**Case presentation:**

We describe a case of neoplasm of the bladder in a 74 years-old Caucasian man presenting with massive hematuria. Based on clinical, instrumental and histological findings a diagnosis of PMM was made. The patient underwent trans urethral resection of bladder tumor plus intravesical Bacillus Calmette–Guérin.

**Conclusions:**

To make a correct diagnosis, clinical history, endoscopic evaluation, histopathological examination and immunohistochemistry, are necessary. Multidisciplinary evaluation is required to discriminate primary from metastatic malignant melanoma.

## Background

Primary malignant melanoma (PMM) of the genitourinary tract represents a very rare clinico-pathologic entity and the urethra represents the most common involved site [1]. To date, less than 50 cases of PMM of the urinary bladder have been reported in the literature [1]. This tumor has been reported to occur over a wide age range with a slightly higher prevalence in men [1]. Although the exact pathogenesis of PMM of the bladder is unknown, some authors have theorized a potential link with bladder melanosis [1]. Typically, PMM of the bladder does not manifest itself until the disease is advanced [2]. Surgery represents first-line treatment option. Based on tumor stage, surgical options include: trans-urethral resection, partial cystectomy or radical cystectomy [2]. Immunotherapy, radiotherapy, and chemotherapy represent additional treatment options [2]. We describe a case of PMM of the bladder in a 74 years-old man presenting with massive hematuria and treated with trans urethral resection of bladder tumor (TURB-T) plus intravesical Bacillus Calmette–Guérin (BCG).

## Case presentation

**Fig. 1:**
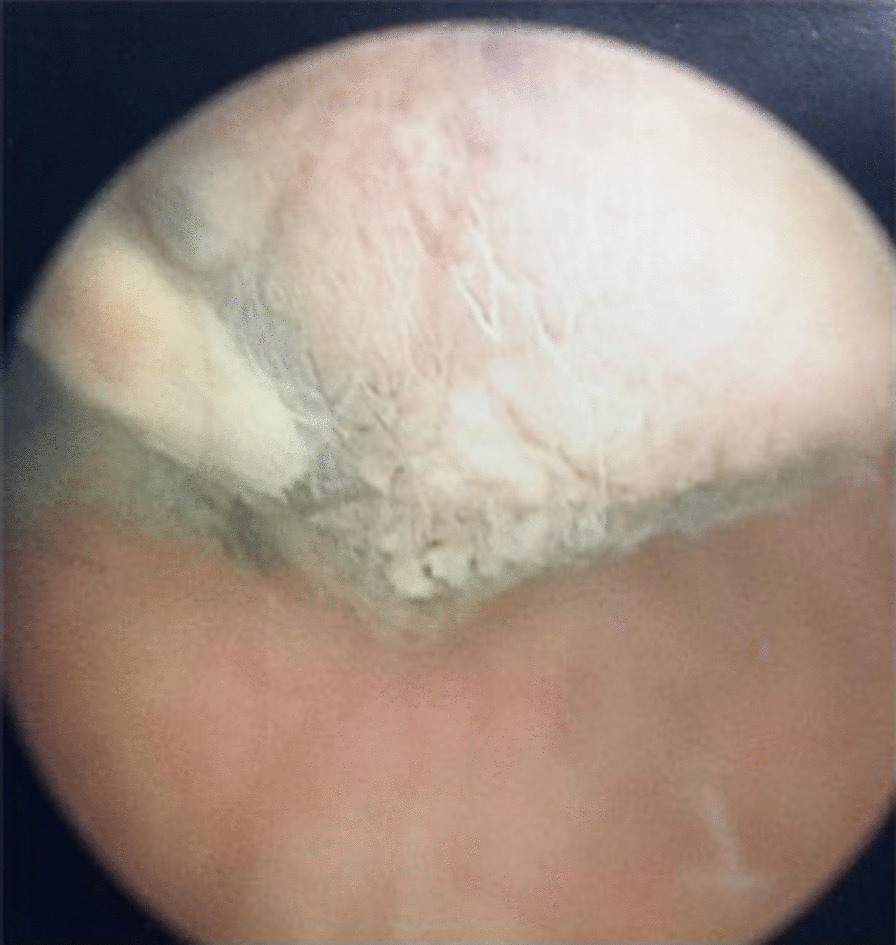
Endoscopic appearance of primary malignant melanoma of the bladder. The tumor appears as a dark pigmented mass

**Fig. 2:**
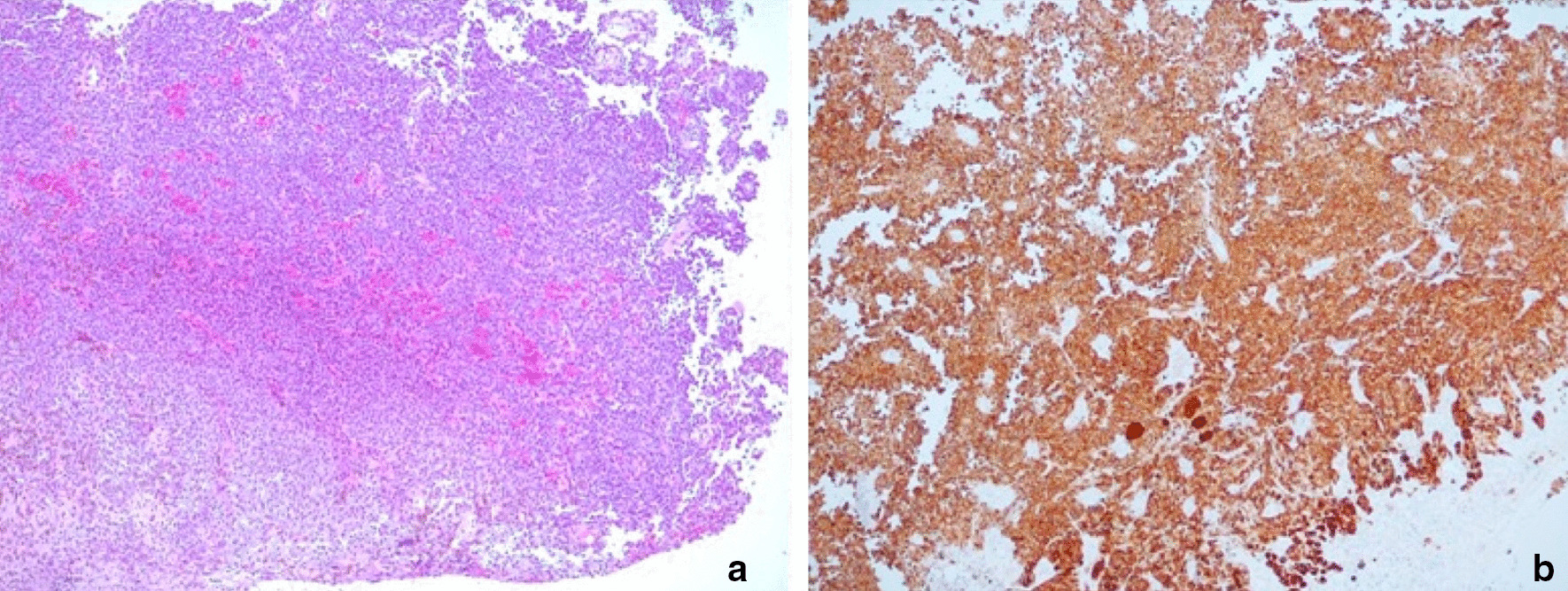
**a** Hematoxylin/eosin staining (50× magnification) showing a proliferation composed of a mixture of spindle and epithelioid cells with abundant cytoplasm, irregular nuclei, prominent eosinophilic nucleoli and severe pleomorphism; **b** Immunohistochemical staining (50× magnification) revealing positivity for S100

A 74-year-old Caucasian man was admitted to the Urology Department for an episode of macrohematuria. His past medical history was only relevant for clear cell renal carcinoma treated with radical nephrectomy 2 years before. On admission, his serum hemoglobin level was 7.5 mg/dl and his glomerular filtration rate was 96 ml/min. Two units of red blood cells were immediately transfused. The patient underwent abdominal ultrasound that revealed a 3 cm hypoechoic lesion involving the left lateral bladder wall. The preliminary cystoscopy revealed an atypical pedicled lesion characterized by a brownish black pigment involving the anterior bladder wall (Fig. 1). The main diagnostic hypothesis was malignant melanoma. The patient was scheduled for TURB-T. Complete resection of the tumor was performed using a standard monopolar resectoscope. The totally resected specimen weighed 40 g. The postoperative course was uneventful. Histologic examination showed a proliferation composed of a mixture of spindle and epithelioid cells with abundant cytoplasm, irregular nuclei, prominent eosinophilic nucleoli and severe pleomorphism. Moreover, heavily pigmented melanosomes and macrophages containing melanine pigment were evident (Fig. 2a). Immunohistochemical study showed positivity for S100 and MART-1/MELAN-A, and negativity for desmin, DE-R-11, GATA3, p63 and cytokeratin 7 and of paired-box 8 (PAX-8) (Fig. 2b). These findings were in line with histopathological diagnosis of malignant melanoma. A post-operative Fluorine-18 fluorodeoxyglucose Positron emission tomography-computed tomography excluded concomitant pathologic foci. Dermatological exam, gastroscopy, coloscopy and an ophthalmologic exam ruled out the suspicious of a secondary lesion from a primitive malignant melanoma elsewhere. The patient was offered a genetic screening but he refused. The clinical stage was T1, N0, M0. Based on institutional multidisciplinary uro-oncologic team evaluation, an adjuvant intravesical BCG treatment was planned. The following schedule was adopted: 6 weekly instillations followed by 3-weekly instillations after 3, 6, 12, 18, 24, 30 and 36 months. At 6 months follow-up both cystoscopy and computerized tomography were negative for recurrence.

## Discussion and conclusion

Typically, melanoma of the bladder can be found in patients with widespread metastatic melanoma originating from the skin. PMM of the bladder is a rare neoplasm that poses diagnostic and therapeutic challenges. Wheelock described the first case of a primary melanoma of the bladder in 1942 [3]. Patient ages range from 7 to 82 years and a slightly higher prevalence in men has been reported. The diagnosis is challenging as presenting symptoms lack of specificity. Indeed, most cases present with hematuria. This was the case for our patient. Other symptoms include dysuria, urgency, nocturia, frequency or urinary retention depending on tumor location and invasiveness. Cystoscopy and transurethral biopsy are the primary method of diagnosis. Endoscopically, the tumor appears as a dark pigmented mass with varying dimensions [4, 5]. Microscopically PMM of the bladder exhibits typical features of melanoma such as nests of large pleomorphic cells with macronuclei and prominent nucleoli. Melanin pigment can be present. However, the histopathological diagnosis of malignant melanoma in the urinary bladder may be challenging and immunohistochemical studies are often required to facilitate the diagnosis [5]. Typically, immunohistochemical evaluations demonstrate positivity for melan-A, and S-100 protein without expression of epithelial markers [4]. However, in some cases, neoplastic melanocytes may express epithelial markers that may lead to an erroneous diagnosis of carcinoma.

It is crucial to discern whether a bladder melanoma is primary or metastatic as metastatic melanoma is much more common than primary tumors. A careful patient history, physical examination of the skin, and evaluation for other visceral primary sites are required to confirm the primary nature of the tumor [5]. In 1976, Ainsworth and colleagues established criteria to differentiate malignant melanoma of primary bladder from metastasis: absence of any previous skin lesion, or cutaneous malignant melanoma, or primary visceral malignant melanoma, recurrence pattern showing consistency with the primary tumor diagnosis, atypical melanocytes at the tumor margin on microscopic examination [4].

Given the rarity of the disease, the best treatment option is not well recognized. A wide range of treatment options have been proposed. TURB-T, partial cystectomy, and radical cystectomy are usually performed as first-line treatment options. In details, TURB-T can be a valid option in localized, small PMM of the bladder. Partial and radical cystectomy are more aggressive treatment options required in patients with more advanced diseases [1, 6, 7]. Adjuvant intravesical administration of BCG has been proposed in some cases [8]. Indeed, transurethral BCG injection has the potential to increase humoral antimelanoma antibody levels in patients with bladder melanoma [9]. However, data are very limited and long-term follow-up is unavailable. The present case further underlines that TURB-T plus intravesical BCG may be a valid option in selected cases. However, the prognosis of PMM of the bladder is generally poor with about two-thirds of the patients dying of metastatic disease within 3 years [5]. Consequently, careful follow-up is mandatory. In consideration of the rarity of the disease and the poor prognosis, PMM of the bladder represents diagnostic and therapeutic challenges. TURB-T plus adjuvant intravesical BCG may potentially represent a reasonable option in selected cases. However wider series with long-term follow-up data are required to confirm these preliminary data.

## Data Availability

Not applicable.

## Abbreviations

BCG: Bacillus Calmette–Guérin
TURB-T: Trans urethral resection of bladder tumor
PMM: Primary malignant melanoma
